# Transition from pediatric to adult care in emerging adults with type 1 diabetes: a blueprint for effective receivership

**DOI:** 10.1186/s40842-019-0078-7

**Published:** 2019-03-06

**Authors:** Jennifer Iyengar, Inas H. Thomas, Scott A. Soleimanpour

**Affiliations:** 1 Diabetes Transition Program, University of Michigan Health System, Ann Arbor, MI USA; 2 Division of Metabolism, Endocrinology & Diabetes and Department of Internal Medicine of the University of Michigan Medical School, 1000 Wall Street, Brehm Tower Suite 5317, Ann Arbor, MI 48105 USA; 3 Division of Endocrinology and Department of Pediatrics and Communicable Disease of the University of Michigan Medical School, Ann Arbor, MI USA

**Keywords:** Transition, Complications, Hemoglobin A1c, Diabetes-related distress

## Abstract

For adolescents and emerging adults, the transition from pediatrics to adult care is fraught with challenges both inside and outside the clinical arena, including assuming independent care for diabetes, working with new adult providers, and overcoming concomitant psychosocial issues, while maintaining work/school-life balance. Not surprisingly, glycemic control in emerging adults with type 1 diabetes is amongst the worst in all age groups. Thus, new and comprehensive strategies are needed by both pediatric and adult diabetes care teams to support young adults during the transition to adult care. In this review, we focus on challenges during the transition period and provide evidence-based recommendations for a receivership model to assist adult diabetes care teams in addressing these concerns. By coordinating efforts with pediatrics providers, identifying strengths and deficiencies in self-care, establishing rapport with young adult patients, directly addressing prevalent psychosocial concerns, and developing a team-based approach to keep patients engaged, adult care teams can prioritize support for the most vulnerable transition patients. Improved strategies to propel emerging adult patients through the transition period towards habits leading to optimal glycemic control could have a major long-term impact on preventing diabetes-related complications.

## Introduction

Effective management of type 1 diabetes (T1D) is a challenge for all patients and care providers due to the intricacies of treatment, proper dietary/exercise balances, attention required for self-monitoring and medical visits, affordability of insulin and diabetes supplies, and challenges related to maintenance of ongoing insurance coverage. These demands are intensified in susceptible patient populations. Indeed, the management of T1D in adolescents and young adults *transitioning* from pediatric to adult care is fraught with challenges. Recent studies indicate that glycemic control substantially declines amongst T1D patients in the age range for transition to adult care (between the ages 18–30) with only 14% of patients meeting hemoglobin A1c targets [1]. Moreover, the risks of diabetes complications amongst transition age patients is already elevated, with nearly one-third of patients having evidence of one early diabetes-related complication [2]. Given the potential for long-term harm from poor glycemic control and diabetes complications in the decades beyond transition, an increasing focus within the pediatrics community is to formally prepare young adults to meet the challenges of transition.

An underappreciated component of the transition process is the responsibility of adult providers to actively participate in the stewardship of young adults through this challenging time. As many young adults delay assuming adult roles until their late 20s, the roles of adult providers in shepherding these patients through the immediate and longer-term phases of “emerging adulthood” are magnified [3]. Thus, we hypothesize that adult providers play an essential “receivership” role that may be similarly critical to those of their pediatric colleagues for successful diabetes transition to adulthood. In light of this hypothesis, we provide a focused review of the evidence elucidating the specific challenges nascent to the transition period and strategies for improving outcomes with 5 suggested areas of focus for adult providers to incorporate into a receivership approach for young adults with T1D.

### Insights into the transition experience

Recently, an increasing number of rigorous studies have begun to identify the challenges observed in patients transitioning from pediatric to adult care. Indeed, many of these studies have been carefully reviewed elsewhere [3–7]. Here we briefly review several key observations that serve to inform and tailor our recommendations for an improved transition receivership model. While patients and families generally report an expectation to transition to adult care between ages 17–19 years [8], several prospective and retrospective studies report the actual mean age of transition to be somewhat later, between 19.5–20.1 years of age, with nearly 80% of patients arriving at adult providers by 21 years [9–11]. Amongst the most important reasons listed by patients to transition to adult care included a sense of being “too old” to remain within pediatrics care, following the suggestion of their pediatrics provider, or beginning their college education [10]. While many pediatrics patients felt prepared for transition, more than one-third did not feel adequately prepared [9]. The patients’ sense of transition preparation also was highly associated with that of transition satisfaction, with approximately 38% of patients feeling less than satisfied with their transition to adult care [10]. While overall glycemic control tends to be worse amongst the transition age group, it is notable that patients with higher hemoglobin A1c concentrations were less likely to transition to adult care at younger ages. Further, patients transitioning to an adult provider have a 2.46-fold elevated risk of poor glycemic control by the time of their first adult visit, with elevations in hemoglobin A1c from 7.5% in their final pediatrics visit to 9.2% by their first adult visit [11]. Taken together, these studies suggest that pediatric care teams play a crucial role in successfully preparing patients for transition; however, additional structured support in the adult care setting is necessary for the large number of patients who retain outstanding concerns and to reinforce the survival skills necessary to avoid complications.

### 5 recommendations for effective receivership

#### *Recommendation #1*: Communicate with pediatric colleagues during transition to coordinate care and minimize gaps

Young adults with T1D often have long-standing relationships with their pediatric providers. Indeed, 64% of T1D patients between the ages of 18–30 who were still in pediatric care noted they had avoided transitioning to adult care due to an emotional attachment to their pediatric provider [9]. During the transition process, not only is the patient entrusting their care to a new and unfamiliar provider, but their pediatric provider needs to feel comfortable that their adult counterpart will provide high quality care. A good working relationship between adult and pediatric providers can aid in navigating the logistical hurdles during the transition process and ensure continuity of care.

Pediatric providers may benefit from objective methods for self-evaluation, thus allowing for guidance during transition preparation. To this end, the National Alliance to Advance Adolescent Health has recognized six core elements of health care transition (HCT), which can be used by an individual provider or practice network to assess their current level of health care transition support (reviewed in [12]). The six core elements of HCT outline recommendations that providers or practices develop programs as follows: **(1)** develop a transition and/or care policy, **(2)** tracking and monitoring of progress, **(3)** transition readiness and/or orientation to adult practice, **(4)** transition planning and/or integration into adult approach to care or practice, **(5)** transfer of care and/or initial visit, and **(6)** transition completion or ongoing care [12]. As T1D bears close similarity to the lifetime needs of other childhood chronic diseases, pediatric (and adult) providers can use these National Alliance tools to ensure their practice meets a broad standard of care applicable for all transition patients.

Appropriate timing of transition is a key consideration to ensure patients are ready to self-manage their diabetes in the adult care environment. While the average age of transition for patients with T1D is 19–20 years old [9–11], recommendations from American Academy of Pediatrics (AAP) suggest that transition planning should begin as early as age 14, including regular assessments to identify self-care needs and goals [13]. The AAP recommendations for pediatric providers build upon the 6 core elements for HCT, suggesting that pediatricians modify their practices to: **(1)** create and discuss transition care with youths and/or families, **(2)** track progress of youths’ and/or families’ transition preparation and transfer, **(3)** conduct transition readiness assessments, **(4)** develop a transition plan (including needed readiness assessment skills and medical summaries, prepare youths for adult approach to care, and communicate with new physicians) **(5)** transfer of care with information and communication including residual pediatric clinician’s responsibility, and **(6)** obtain feedback on the transition process and confirm young adults have been seen by new physicians [13].

While several previous studies and guidelines provide recommendations for pediatric diabetes providers to prepare patients for transition [3, 13–15], it is notable that there is wide variation in transition care for young adults with T1D [16]. With these guidelines in mind, pediatric diabetes providers should consider preparing young teens with T1D for their eventual transition to adult care by focusing at least yearly on broad educational goals, including the knowledge of general goals of T1D management, expertise in the skills necessary for T1D self-care (such as for blood glucose monitoring, insulin use, hypoglycemia management/prevention), showing the ability to arrange medical care, and an understanding of what to do in case of emergency or when feeling ill [3]. Frequent discussions regarding transition at an early age will allow pediatric providers many opportunities to evaluate the teens’ mastery of these concepts, which then could shape future conversations regarding the transition to adult care. Indeed, there is no “one-size fits all” time to transition, rather the decision to transfer to adult care should be made with input from the pediatric provider, patient, and caregiver(s) reflecting individual patient factors including self-management skills and psychosocial considerations.

Among patients age 18–30 years who had recently established care with an adult provider, the most common reason for transitioning was on the suggestion of the pediatric provider [9]. Sixty-three percent of patients reported receiving a recommendation for an adult provider. In a study of patients with T1D who had recently transitioned to adult care in Germany, patients reported that the two most helpful ways to improve transfer outcomes was to provide recommendations for possible new physicians and then provide information about these physicians [17]. Similar results have been found in the United States with patients noting the importance of having a recommendation for a specific adult provider [9, 10, 18].

As an adult provider, it can be beneficial to be identified to pediatric colleagues as a provider with an interest in transition care. This can help pediatric providers appropriately pair their patients with an adult provider who understands the unique needs of this population and who collaborates on care coordination. Since over half of the patients in this age group use an insulin pump for glycemic management [1], adult providers working in this population should be well-versed in a range of insulin pumps as well as continuous glucose monitoring systems. Having the patient meet with the new adult provider before transition is an appealing option to ensure a good match before making the switch. However, data from the T1D Exchange suggest that this only occurs in 18% of cases [9] and may not always be feasible especially in practices where pediatric and adult clinics are held in separate locations.

Despite the emphasis on a coordinated transition process, gaps in care are still common. Twenty-one percent of patients had a gap of > 6 months during the move from pediatric to adult care [9]. Patients who felt less prepared for transition were more likely to have a gap [9, 10], suggesting that these patients may be more vulnerable to lapses in care. Patients identify care coordination between pediatric and adult providers as a key issue during the transition process [8]. We recommend that provider teams coordinate the timing of the last pediatric visit with the first adult visit to ensure patients maintain a standard quarterly appointment schedule without interruption. If the patient cannot get a new appointment with their chosen adult provider in a timely manner, an additional pediatric visit should be scheduled in the interim, and an adult provider visit can be scheduled 3 months from that appointment. This again highlights the importance of coordinated care between adult and pediatric diabetes teams with reinforcement from pediatric providers for patients that are uneasy during the transition process.

#### *Recommendation #2:* Objective assessment of knowledge and skills levels can help the adult provider capitalize on strengths and identify needs for intervention

A variety of readiness tools have been developed for young adults with chronic conditions to provide an objective assessment of whether patients have the knowledge and skills needed to self-manage their disease [19]. Some tools, such as the TRxANSITION scale, Transition Readiness Assessment Questionnaire (TRAQ), and the Self-Management Skills Assessment Guide (SMSAG) are generic assessments designed for broad applicability with a range of chronic health conditions [20–22]. Common themes in these assessments include understanding of their health condition, medication management, interaction with providers, and management of daily activities. Only a few tools have been specifically designed and are actively being tested for T1D transition, most notably the Readiness assessment for Emerging Adults with Diabetes Diagnosed in Youth (READDY) [23]. The advantage of a disease-specific tool is to provide a more detailed assessment of specific diabetes management skills including carbohydrate counting, insulin injections, insulin pump management, interfacing with insurance providers, and sick day rules.

For adult providers, a potential pitfall is to presume that assessment of transition readiness falls solely on pediatric providers. Assessment of self-management skills is an ongoing process and one that continues after the patient has established care with an adult provider. Data from the T1D Exchange indicate variability among transition preparedness topics; 86% of participants discussed managing diabetes on their own with their pediatric providers, while 80% discussed screening tests, 60% discussed alcohol use in diabetes, and only 33% discussed reproductive health issues [9]. Despite ideal transition preparedness, individual patients will have aspects of their management where they feel confident and aspects where they need ongoing reinforcement. Adult providers may choose to administer transition preparedness questionnaires during the first appointment with the patient, review transition preparedness materials from the pediatrics provider, or collaborate with diabetes educators to help identify and fill in these gaps. Assessment of diabetes-related skills may also provide an opportunity to re-educate patients based on identified deficits and allow providers to enact new treatment modalities at a time when young adults are highly engaged [24, 25].

#### *Recommendation #3:* Focus on establishing a relationship, not just perfecting A1c

Data from the T1D Exchange indicate that glycemic control during the young adult period is poor, with higher hemoglobin A1c levels than in any other age group [1]. Hemoglobin A1c levels peak around age 19 at an average of 9.2%, then trend down gradually until age 30 before plateauing between 7.5–7.8%. Only 14% of registry patients between the age of 18–25 met the American Diabetes Association target hemoglobin A1c of < 7% [1]. While tight glycemic control remains the central tenet of T1D management [26], the management of young adults with T1D requires striking a balance between expectations for tight glycemic control and maintaining patient engagement if control is suboptimal. Patients reported one of the positive aspects of transitioning to adult care is the autonomy in decision-making [27]. Patients also highlighted the advantages of an adult providers’ willingness to collaborate with them (rather than their parents/caregivers) to find solutions and hold them accountable [27]. However, patients in this age group report difficulty meeting stricter hemoglobin A1c goals and challenges with adherence [8]. While young adult patients desire increasing independence, it is imperative that adult diabetes providers are aware of the challenges of diabetes management in this age group and to have expectations tailored accordingly [8].

A good patient-provider working relationship establishes the foundation needed to work on improving glycemic control and preventing complications in the future. Large studies addressing the post-transition experience are lacking, but a recent single-center study suggests that post-transition patients with high satisfaction reported that their new provider earned their confidence, listened carefully, and involved them in management decisions [8]. The adult diabetes literature suggests that strong patient-provider relationships can have a positive impact on diabetes self-care and glycemic control [28–30]. Hence, an initial focus on building good rapport with transition patients may be a key to successful long-term glycemic control.

#### *Recommendation #4*: Develop a strategy to routinely identify and address psychosocial needs for young adult patients

Psychosocial issues are common in patients with diabetes and can lead to poor outcomes if left unaddressed. In adults with diabetes, depression is nearly twice as common as compared to non-diabetic adults [31]. The estimated prevalence of depression correlates with gender and is higher in women with diabetes (28%) than men (18%) [31]. In adolescents, the prevalence of depressive symptoms varies widely, with studies reporting rates from 11.3–33% [32–35]. The largest of these studies assessed 2672 youth aged 10–21 from the SEARCH for Diabetes in Youth Study and found that 14% reported mildly depressed mood on the Center for Epidemiologic Studies Depression Scale (CES-D), while 8.6% reported moderate or severe symptoms [34]. Similar to studies in adults, adolescent females and those with T2D were more likely to report depressive symptoms [33, 34]. Depression, however, is not the only mental health concern in this population. Adolescents with T1D have been found to have significantly higher rates of anxiety [32, 36], eating disorders [32, 37–39], and diabetes-related distress [40].

Addressing these issues is vital due to the effects on diabetes-related outcomes. Depressive symptoms and other mental/emotional health concerns in adolescents with diabetes are associated with decreased frequency of self-monitoring of blood glucose [33, 36], higher hemoglobin A1c concentrations [32, 36, 41], increased frequency of emergency department visits [34], and increased risk of hospitalization [35]. Data from the adult population are concordant with those of adolescents but also identified an association between depressive symptoms and risk of microvascular and macrovascular complications [42].

The American Diabetes Association (ADA) recommends integrating psychosocial care as part of the medical management of diabetes, including routine depression and diabetes distress screening, particularly in patients not meeting treatment targets or with diabetes complications [43]. The ADA also recommends screening for anxiety and disordered eating behavior in patients exhibiting suggestive signs or symptoms. Few interventions have been studied specifically in the T1D transition population. A study of 15 T1D patients ranging in age from 18 to 30 years found that participation in a professionally-led support group reduced diabetes distress [44]. Another study of 77 patients, ages 12–20 years with T1D randomized to coping skills training or to a control group, found that those who participated in the coping skills training had significantly lower HbA1c levels after 12 months [45]. However, this study did not focus solely on patients with psychosocial needs at enrollment. Patients with symptoms of depression, distress, eating disorders, or anxiety should be promptly referred to an experienced mental health provider well-versed in these conditions, particularly those for whom mental health symptoms may be adversely affecting diabetes management.

#### *Recommendation #5*: A team-based approach can help young adults stay engaged

Providing high quality diabetes care during the transition process may benefit from a team-based approach. This may include collaboration from physicians, advanced practice providers, nurses, certified diabetes educators, registered dieticians, social workers, medical assistants, administrative staff, and even from the patients and families themselves. Formation of a team-based approach for transition care would allow providers to develop a structured transition plan to best deploy local resources and address needs specific to their patient population, including consultation for insurance- or financial-related concerns that may preclude optimal care. While it is unlikely that a transition patient will need to meet with each member of the team at every visit, we recommend formally introducing patients to the team at the initiation of transition to adult care to avail them of the totality of resources within their transition experience.

There are insufficient data to suggest the superiority of any single transition model. However, there are some data to suggest that having a care navigator as an identified point-of-contact for patients can reduce drop-out rates after transition [46]. One study found that use of a transition coordinator as part of a comprehensive transition program led to reduced HbA1c levels when compared to an unstructured program [47]. In addition to being a primary contact person for the patient, a care navigator can track patients, rebook missed appointments, and reach out if patients are lost to follow-up. They can also identify barriers to care and link the patient to other members of the team to overcome specific concerns. Further, a care navigator can assist the patient with identifying additional care providers, in the event mental health or financial issues were previously identified by the pediatric provider.

Another important, yet often overlooked, resource are the patients and families themselves. Those that have been through the transition process can provide valuable patient-centric feedback and identify areas for quality improvement. It may be appropriate to formalize this feedback together with a patient advocate who can share insights about the patient experience and partner with the team to provide the best possible care.

## Conclusions

**Fig. 1:**
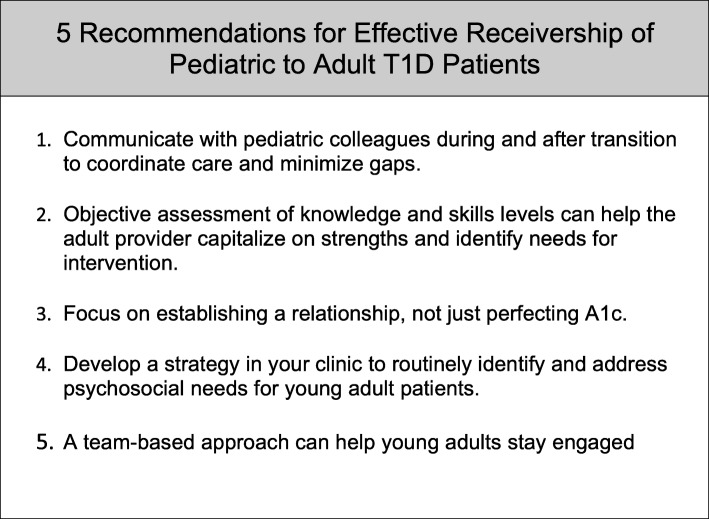
Summary of the five recommendations for effective receivership

Here we identify the pervasive challenges that are integrally involved in the transition of young adult patients with T1D from pediatrics to adult care and provide several evidence-based recommendations to optimize the transition process (Fig. 1). By developing a transition environment predicated on coordination with pediatrics colleagues, relationship-building with T1D patients, objectively identifying areas for intervention, assessing psychosocial needs, and using a cooperative approach, we believe that adult providers can avoid pitfalls that ultimately lead young adults away from a lifetime focus on diabetes care and reduce the risks of diabetes complications. Finally, adult providers should consider closing the transition loop by communicating back to pediatrics colleagues regarding outcomes with their patients. It is evident that additional studies will be needed to test these approaches and determine potential long-term benefits for transition patients with T1D. Despite the need for further evidence, it is clear that a strong cooperative focus on transition by both the pediatric and adult diabetes care community will no doubt inspire all parties (providers, support staff, families, and the patients themselves) to continue to develop innovative methods to improve glycemic control and avoid long-term complications.

## Abbreviations

AAP: American Association of Pediatrics
ADA: American Diabetes Association
CES-D: Center for Epidemiologic Studies Depression Scale
HCT: Health Care Transition
SMSAG: Self-Management Skills Assessment Guide
T1D: Type 1 Diabetes
TRAQ: Transition Readiness Assessment Questionnaire
